# Quantifying maternal investment in mammals using allometry

**DOI:** 10.1038/s42003-024-06165-x

**Published:** 2024-04-18

**Authors:** Tim E.R.G. Huijsmans, Alexandre Courtiol, Ann Van Soom, Katrien Smits, François Rousset, Jella Wauters, Thomas B. Hildebrandt

**Affiliations:** 1 Department of Internal Medicine, Reproduction and Population Medicine, Faculty of Veterinary Medicine, Ghent University, Salisburylaan 133, 9820 Merelbeke, Belgium; 2 Department of Evolutionary Genetics, Leibniz Institute for Zoo & Wildlife Research, Alfred-Kowalke-Str. 17, 10315 Berlin, Germany; 3 Institute of Evolutionary Science of Montpellier, University of Montpellier, CNRS, IRD, campus Triolet, 34095 Montpellier cedex 05, France; 4 Department of Reproduction Biology, Leibniz Institute for Zoo & Wildlife Research, Alfred-Kowalke-Str. 17, 10315 Berlin, Germany; 5 Laboratory of Integrative Metabolomics, Department of Translational Physiology, Infectiology and Public Health, Faculty of Veterinary Medicine, Ghent University, Salisburylaan 133, 9820 Merelbeke, Belgium; 6 Department of Reproduction Management, Leibniz Institute for Zoo & Wildlife Research, Alfred-Kowalke-Str. 17, 10315 Berlin, Germany; 7 Freie Universität Berlin, Kaiserswerther Str. 16-18, 14195 Berlin, Germany

**Keywords:** Evolutionary developmental biology, Animal physiology

## Abstract

Maternal investment influences the survival and reproduction of both mothers and their progeny and plays a crucial role in understanding individuals’ life-history and population ecology. To reveal the complex mechanisms associated with reproduction and investment, it is necessary to examine variations in maternal investment across species. Comparisons across species call for a standardised method to quantify maternal investment, which remained to be developed. This paper addresses this limitation by introducing the maternal investment metric – *MI* – for mammalian species, established through the allometric scaling of the litter mass at weaning age by the adult mass and investment duration (i.e. gestation + lactation duration) of a species. Using a database encompassing hundreds of mammalian species, we show that the metric is not highly sensitive to the regression method used to fit the allometric relationship or to the proxy used for adult body mass. The comparison of the maternal investment metric between mammalian subclasses and orders reveals strong differences across taxa. For example, our metric confirms that *Eutheria* have a higher maternal investment than *Metatheria*. We discuss how further research could use the maternal investment metric as a valuable tool to understand variation in reproductive strategies.

## Abstract (web summary)

This study introduces a standardized method to quantify maternal investment: the maternal investment metric for mammalian species, derived from the allometric scaling of litter mass at weaning age by adult mass and investment duration .

## Introduction

In mammals, maternal investment, i.e. the allocation of resources to meet the basic needs of offspring during the pre- and post-natal development, holds a significant influence over the survival and reproduction of mothers and their progeny^1^. The concept of maternal investment has thus played a pivotal role in the development of life-history theory^2^ and population ecology^3^. Understanding the intricate relationships associated with reproduction and maternal investment is essential for comprehending how an animal can achieve adequate fitness within a specific niche^4^.

Maternal investment is shaped by genetically influenced factors connected to life-history traits such as metabolic rate, body mass, and longevity, which all scale with each other due to their relationship with body size^5,6^. In addition, mothers adjust their investment depending on circumstances, such as their condition^7^, access to resources^8^ and experience^9,10^, as well as environmental cues^11^. To gain a comprehensive understanding of these reproduction strategies, it is essential to examine the variations in maternal investment across species that are not simply due to allometry, i.e. due to differences ultimately resulting from variation in body size. Previous studies have tried to compare investment strategies using proxies such as calorimetry, basal metabolic rate, growth rate, and gestation or lactation duration^12–18^. Moreover, experiments within single species (typically insects) have been conducted to understand specific mechanisms^8,19^. However, these approaches have been limited by the scarcity of data and the absence of a standardised methodology capable of comparing a large number of species based on the available information. Consequently, conducting large-scale studies to unravel the complexities of investment strategies has remained unfeasible.

In an effort to overcome this lack, we build on the scaling laws governing life-history traits to propose a metric of maternal investment comparable across mammalian species. Stemming from the allometric concept pioneered by D’Arcy Thompson and Julian Huxley, these scaling laws indicate that biological traits change as a function of an organism’s body size^20,21^. This idea has undergone thorough examination in the realm of mammalian biology, showing consistent patterns across diverse species. Guided by this principle, we introduce the maternal investment metric which we define in terms of the nutritional investment of the mother in her offspring. This innovative tool should facilitate comprehensive comparisons of maternal investment strategies across mammalian species. By utilising empirical data on weaning mass, litter size, adult mass, and investment duration (i.e. gestation + lactation duration) from a comprehensive database encompassing hundreds of mammalian species, we studied the robustness of this metric to alternative regression methods used to fit allometric relationships and to alternative proxies used to quantify the body mass of individuals in a species. We also used the database to study the extent to which taxonomy influences maternal investment once allometry is accounted for.

The chief aim of this paper is to establish a standardised method for comparing and enhancing our understanding of investment strategies among different species of mammal. We designed a tool to comprehensively analyse the maternal investment patterns across species, in order to understand what influences reproductive success and to provide insights for future investigations into reproductive strategies and their consequences.

## Results

### A metric to quantify maternal investment

#### Predictor for maternal investment

Maternal investment in mammals starts with the development of a fertilised oocyte and culminates with a weaned individual. Therefore, the mass at weaning reflects maternal investment until that point. Since litter weaning mass encompasses the entire litter’s mass (i.e. the cumulative body mass measured at weaning age across all offspring from a given litter at weaning age) and thus represents the complete investment by the mother, we considered this variable as the most appropriate predictor to use for the characterisation of offspring mass. To identify whether the litter mass at weaning is correlated with the adult mass and could therefore be allometrically scaled, we measured a linear Pearson correlation between the (log_10_) adult mass and (log_10_) litter weaning mass. This analysis revealed a strong correlation between the litter weaning mass and adult mass (r = 0.967, *N* = 1041, *p* < 0.001). We therefore went on to allometrically scale the litter weaning mass with adult mass.

#### Sensitivity of the allometric relationship to the fitting method

There is a long-standing debate about how to best fit allometric relationships^22–24^. We thus relied on six alternative regression models to estimate the litter mass at weaning. Specifically, we used a simple linear regression (SLR), a linear (heteroscedastic) mixed-effects model accounting for phylogenetic inertia (PLMM), a standardised major axis regression (SMA), a major axis regression (MA), a multiple linear regression model representing an extension of the model SLR (MSLR), and a multiple linear regression representing an extension of the model PLMM (MPLMM). On top of the effect of the adult mass on the litter mass at weaning considered in the bivariate models (SLR, PLMM, SMA and MA), the MSLR and MPLMM models also account for a possible effect of investment duration. The rationale behind these multiple linear regression models (i.e. MSLR and MPLMM) is to account for the possibility that an extended maternal investment in offspring could result in greater offspring growth. This possibility is not a priori obvious since the effect of the investment duration may already be accounted for in the simpler models, via the adult mass predictor, due to the allometric relationship between body mass and the pace of life. The rationale behind the models PLMM and MPLMM is that, for a given adult mass, the litter mass at weaning may differ between taxa due to the influence of factors correlated with phylogeny such as the genetic make-up of individuals or their ecology. Therefore, accounting for the phylogenetic distances between species while fitting the allometric relationship decreases the impact of variation in the density of observations along the mammalian phylogenetic tree on estimates produced by regression methods.

**Fig. 1:**
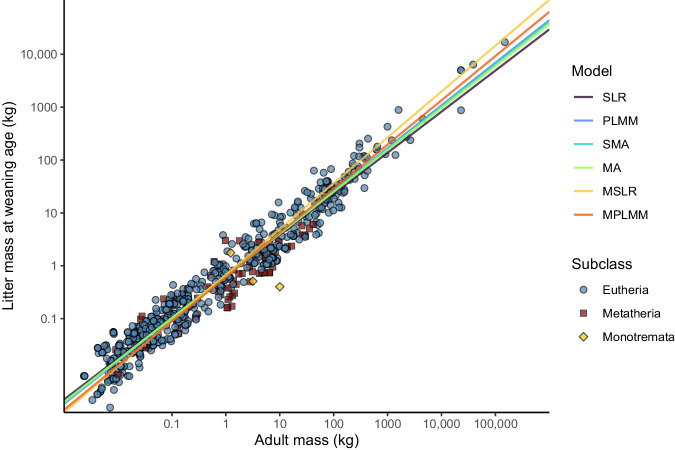
Allometric relationship between adult mass and litter weaning mass in mammals: comparative analysis of six regression models. Scatter plot of the litter weaning mass versus the adult mass in mammals (*N* = 738 species). The scatter plot shows a clear allometric relationship between adult mass and litter mass at weaning age. The six lines represent the predictions stemming from six different regression models (SLR simple linear regression, PLMM phylogenetic linear mixed model, SMA standardised major axis regression, MA major axis regression, MSLR multiple linear regression, MPLMM multiple phylogenetic linear mixed model). For MSLR and MPLMM, the regression lines consider the mean (log_10_) investment duration for all species.

We fitted the linear regressions on a single subset of our data (*N* = 738) that, for each species, contained information about the litter mass at weaning, the adult body mass, the investment duration and phylogenetic position^25^. The estimates of the allometric relationship obtained for the different regression methods are depicted in Fig. 1 and summarised in Table 1.

**Table. 1:** Intercepts and allometric scaling coefficients of six different linear regression models fitted to the same dataset of mammalian species (*N* = 738)

Model	Intercept (CI_95%_)	Scaling *m*_a_ (CI_95%_)	Scaling *d* (CI_95%_)
SLR	−0.196 (−0.216, −0.176)	0.778 (0.765, 0.791)	–
PLMM	−0.192 (−0.534, 0.173)	0.806 (0.782, 0.833)	–
SMA	−0.190 (−0.210, −0.170)	0.799 (0.786, 0.813)	–
MA	−0.192 (−0.212, −0.172)	0.795 (0.781, 0.808)	–
MSLR	0.664 (0.508, 0.819)	0.867 (0.847, 0.887)	−0.398 (−0.470, −0.327)
MPLMM	0.204 (−0.255, 0.628)	0.836 (0.804, 0.870)	−0.199 (−0.334, −0.0701)

*m*_a_ represents the default adult mass and *d* the investment duration. All regression methods led to a good adjustment of the data (r^2^ > 0.947, *p* < 0.001). Note that intercepts given here correspond to the estimates directly given by model fits. They thus correspond to the location where the regression line would cut the log_10_-transformed y-axis.

Using the six fitted models, the expected litter mass at weaning for a given species can be predicted based on the adult mass, and in case of the multiple linear regression models, based on the adult mass and investment duration, for that species. By comparing the actual litter mass at weaning to the predicted litter mass at weaning, a metric can be produced to allow for the objective comparison of maternal investment between species. We propose the following maternal investment metrics based on the ratio between these quantities: ${\it{{MI}}}={\log}_{10}\frac{{observed\; litter\; mass\; at\; weaning}}{{litter\; mass\; at\; weaning\; predicted\; by\; allometry}}$, where *MI* is the maternal investment (a unitless number since [kg]$\times$[kg]^−1^ = [1]). The six maternal investment metrics stemming from our six linear regression models are presented in Table 2.

**Table. 2:** Overview of the maternal investment metrics in mammals according to six alternative regression models fitted to the same dataset (*N* = 738 species)

Model	Metric
SLR	${{MI}}_{{\rm SLR}}={\log }_{10}\frac{{m}_{{\rm lw}}}{{{0.637\times m}_{\rm a}}^{0.778}}$
PLMM	${{MI}}_{{\rm PLMM}}=\log_{10}\frac{{m}_{{\rm lw}}}{{{0.643\times m}_{\rm a}}^{0.806}}$
SMA	${{MI}}_{{\rm SMA}}={\log }_{10}\frac{{m}_{{\rm lw}}}{0.645\times {{m}_{\rm a}}^{0.799}}$
MA	${{MI}}_{{\rm MA}}=\log_{10}\frac{{m}_{{\rm lw}}}{0.643\times {{m}_{\rm a}}^{0.795}}$
MSLR	${{MI}}_{{\rm MSLR}}={\log }_{10}\frac{{m}_{{\rm lw}}}{4.61\times {{m}_{\rm a}}^{0.867}\times {d}^{-0.398}}$
MPLMM	${{MI}}_{{\rm MPLMM}}=\log_{10}\frac{{m}_{{\rm lw}}}{1.60\times {{m}_{\rm a}}^{0.836}\times {d}^{-0.199}}$

m_lw_ represents the observed litter mass at weaning. See legend Table 1 for more details.

The *MI* metrics directly correspond to the residuals associated with the regression lines shown in Fig. 1. For bivariate models using only a single predictor (adult mass), a species characterised by *MI* value of 0 is thus a species that does not deviate from the regression line for the allometric relationship between the litter mass at weaning, the adult mass, and in case of MSLR and MPLMM the investment duration. More generally, an *MI* value of 0 corresponds to a species for which the litter mass at weaning equates to what is expected for an average species with the same adult mass and (if applicable) the same investment duration. A species with a positive or negative *MI* value thus corresponds to a species for which the litter mass at weaning is respectively higher or lower than predicted from a given regression model. Computing the *MI* for all 738 species for which the default adult mass, the investment duration, and the position of any given species within the phylogenetic tree were known resulted in species-level predictions relatively consistent across the six regression methods. Models not accounting for phylogenetic inertia resulted in *MI* estimates similar between each other (all r > 0.92, Quade test: F_3,2211_ = 0.642, *p* = 0.59). However, the fit of the phylogenetic linear mixed models (i.e. models PLMM and MPLMM) revealed a strong phylogenetic signal in the data that is not captured by the allometric relationships. Indeed, both phylogenetic linear mixed models fitted the data significantly better than their non-phylogenetic counterparts (PLMM vs SLR: Likelihood Ratio Test (LRT) = 933, df = 3, *p* < 0.001; MPLMM vs MSLR: LRT = 827, df = 3, *p* < 0.001).

In the model MPLMM, the confidence interval for effect of investment duration (as represented by the scaling coefficient of the allometric relationship between investment duration and the litter mass at weaning) was significantly negative (Table 1). This does not imply that a longer investment duration means a lower maternal investment. In fact, if investment duration becomes the sole predictor in the model, the effect is clearly positive (scaling d = 1.82; CI_95%_ = 1.60, 2.04; *N* = 738). The negative effect of the log_10_ investment duration in the model MPLMM instead implies that the longer mothers invest in their offspring the less the litter mass at weaning increases with adult mass. Together, the adult mass and the investment duration therefore predicted maternal investment significantly better, leading to a more meaningful denominator for the *MI* value. For these reasons, we chose to perform all subsequent steps of our analysis using the model MPLMM, which captures both the effect of the phylogeny and that of the investment duration.

#### Sensitivity of the allometric relationship to adult mass definition

**Fig. 2:**
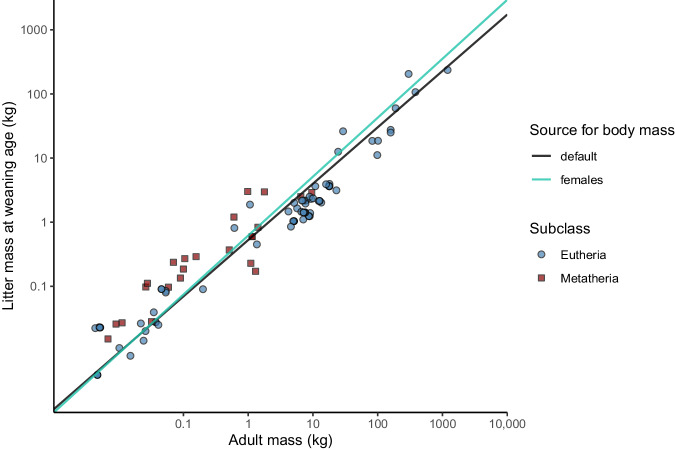
Comparing the allometric relationship between two proxies for adult mass and litter mass at weaning age. Scatter plot of the litter mass at weaning versus the adult mass in a subset of mammals for which both the default adult mass and the female adult mass were available (*N* = 105 species). The scatter plot shows that the allometric relationship between adult mass and litter mass at weaning age differed little depending on the adult mass proxy considered. Darker points are the result of overlapping observations for different species. The two regression lines represent the predictions stemming from a multiple phylogenetic linear mixed model (MPLMM).

**Fig. 3:**
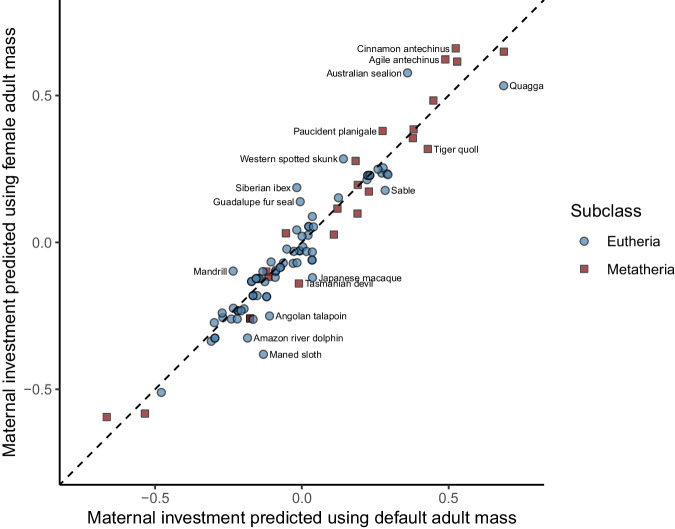
Maternal investment predictions from a multiple phylogenetic linear mixed model using two different proxies for adult mass. Scatter plot of the maternal investment metric (MI_MPLMM_) in mammals predicted using default or female adult mass (*N* = 105 species). Predictions stem from a multiple phylogenetic linear mixed model (MPLMM) fitted on two alternative proxies of adult mass (default or female). The species name is given for observations for which the difference in maternal investment estimates is greater than 0.1. The dashed line represents the perpendicular bisector (i.e. line of equation *y* = *x*).

The first series of models was based on the default adult mass (i.e. computed by the database provider – Amniote^26^ – as “the body mass of an adult individual in grams”, not taking sex into account). Since, in mammals, the maternal investment should be related to the body mass of the female specifically, we used a subset of the data for which estimates of adult mass were available for both sexes (*N* = 105) and refitted the model MPLMM separately for the two proxies for adult mass (Fig. 2). The predicted maternal investment metrics remained relatively similar for the same species irrespective of the type of adult mass proxy used (exact Wilcoxon-Pratt signed-rank test, z = 1.66, *p* = 0.0970). The difference between the maternal investment metric computed using the default adult mass and the maternal investment metric computed using the female adult mass differed by more than 0.1 for only 15.2% of the species (Fig. 3). A difference of 0.1 in maternal investment accounts for ca. 0.40 SD in the variation in the *MI* metrics. These results demonstrate that the choice of the adult mass proxy had little influence upon inferences on maternal investment across a wide range of mammals. This finding justifies our decision to analyse default adult mass rather than female adult mass, as it allows us to benefit from a much larger sample size.

### Taxonomic patterns of maternal investment

Since the fit of the phylogenetic linear mixed models (PLMM and MPLMM) revealed a strong phylogenetic signal that is not captured by the allometric relationship, we now turn to the study of how the taxonomy impacts the maternal investment metric.

#### Effect of mammalian subclasses on maternal investment

**Fig. 4:**
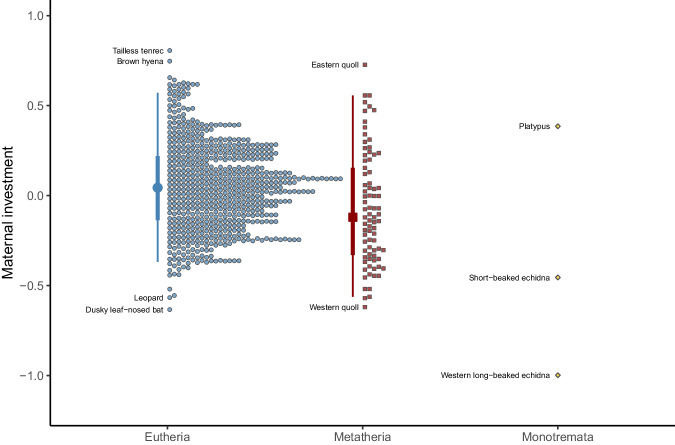
Distribution of the maternal investment metric across mammalian subclasses. Distribution of the maternal investment metric (MI_MPLMM_) across mammalian subclasses (*N* = 738 species). The thin vertical lines delineate 95% of the central observations, and the thick vertical lines 50%. The large symbols in the centre of the vertical lines represent the median values. This information is not provided for *Monotremata* due to the low sample size for this subclass (*N* = 3). The smaller symbols located right from the vertical lines indicate individual species, with *y*-values corresponding to the predicted maternal investment. These symbols are spread out along the *x*-axis for the purpose of visualisation. The maternal investment of *Eutheria* was significantly higher compared to the maternal investment of *Metatheria*.

Using the introduced maternal investment metric (*MI*_MPLMM_), we compared the maternal investment across the three mammalian subclasses using the same 738 species that we used for the comparison of fitting methods (Fig. 4). This resulted in a mean ± SD maternal investment of 0.0483 ± 0.242 for *Eutheria* (*N* = 654), −0.0708 ± 0.313 for *Metatheria* (*N* = 81) and −0.356 ± 0.697 for *Monotremata* (*N* = 3). A Kruskal-Wallis test revealed a significant difference in maternal investment between subclasses (Kruskal–Wallis test: χ^2^ = 16.2, df = 2, *p* < 0.001). A post-hoc comparison showed that the maternal investment of *Eutheria* was significantly higher compared to that of *Metatheria* (asymptotic Wilcoxon–Mann–Whitney test; z = 3.87, *p* < 0.001). The low sample size for *Monotremata* precludes meaningful pairwise comparison with this group. The three species of this subclass included in our analysis widely differed in maternal investment estimate. While the Platypus (*Ornithorhynchus anatinus*) was associated with a relatively high *MI*_MPLMM_ value, one species of echidna (the Western long-beaked echidna – *Zaglossus bruijnii*) presented the smallest maternal investment value of all 738 species analysed.

Refitting the model MPLMM so as to account for the mammalian subclass shows that the scaling coefficients of the allometric relationships significantly differed between *Eutheria* and *Metatheria* (LRT of interaction “subclass”:“log_10_ default adult mass” = 7.22, df = 1, *p* = 0.023; LRT of interaction “subclass”:“log_10_ investment duration” = 7.50, df = 1, *p* = 0.014). Consequently, we refitted the model MPLMM independently in *Eutheria* and *Metatheria* to obtain parameters required to establish subclass-species *MI* formulas. For *Eutheria*, we obtained a scaling coefficient for the default adult mass of 0.848 (CI_95%_ = 0.818, 0.865) and a scaling coefficient for the investment duration of −0.190 (CI_95%_ = −0.217, −0.0373). For *Metatheria*, we obtained a scaling coefficient for the default adult mass of 0.778 (CI_95%_ = 0.686, 0.845) and a scaling coefficient for the investment duration of −0.0806 (CI_95%_ = -0.404, 0.279). Omitting the investment duration, and thus refitting the model PLMM, yielded a scaling coefficient for the default adult mass of 0.820 (CI_95%_ = 0.802, 0.840) for *Eutheria* and 0.748 (CI_95%_ = 0.703, 0.811) for *Metatheria*.

#### Effect of mammalian orders on maternal investment

**Fig. 5:**
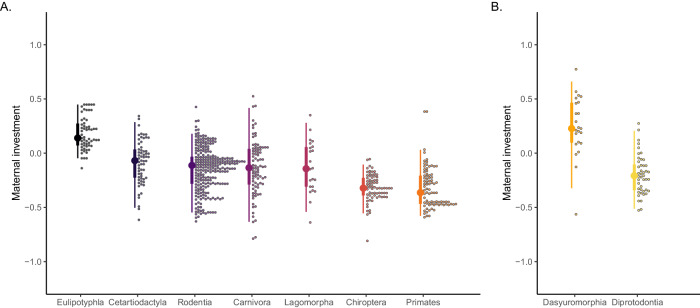
Distribution of the maternal investment metric across *Eutherian* and *Metatherian* orders. Distribution of the maternal investment metric (*MI*_MPLMM_) across *Eutherian* orders (**A**) and *Metatherian* orders (**B**). Note that y-axis values should not be compared across orders from different mammalian subclasses (i.e. across panels) since they stem from different fits of the model MPLMM. See legend of Fig. 4 for other details.

Using the maternal investment metrics (*MI*_MPLMM_) computed separately for the 632 *Eutherian* species and for the 67 *Metatherian* species, we compared the maternal investment across mammalian orders within these mammalian subclasses (Fig. 5). The data used here correspond to the subset for which all required information was available for more than 15 species per order. Maternal investment metrics significantly differed between orders within *Eutheria* (Kruskal–Wallis test: χ^2^ = 196, df = 6, *p* < 0.0001) and within *Metatheria* (exact Wilcoxon–Mann–Whitney test: z = 5.48, *p* < 0.001). Within *Eutheria*, *Eulipotyphla* (*N* = 58) had the highest average *MI* value (0.183 ± 0.152) and *Primates* (*N* = 83) the lowest one (−0.318 ± 0.191). Yet, considerable variation was present within orders. For example, variation in *MI* seems particularly large in *Carnivora* (−0.134 ± 0.258; *N* = 85).

Refitting the model MPLMM so as to account for the mammalian orders shows that the scaling coefficient of the allometric relationship between the default adult mass and the litter mass at weaning tended to significantly differ between mammalian orders within both subclasses (LRT of interaction “order”:“log_10_ default adult mass”: *Eutheria* = 21.6, df = 6, *p* = 0.0571; *Metatheria* = 16.6, df = 1, *p* = 0.036). The results were however less clear for the allometric relationship between the investment duration and the litter mass at weaning (LRT of interaction “order”:“log_10_ investment duration”: *Eutheria* = 12.5, df = 6, *p* = 0.134; *Metatheria* = 1.61, df = 1, *p* = 0.314).

### Illustration of the maternal investment metric using 20 representative mammalian species

**Fig. 6:**
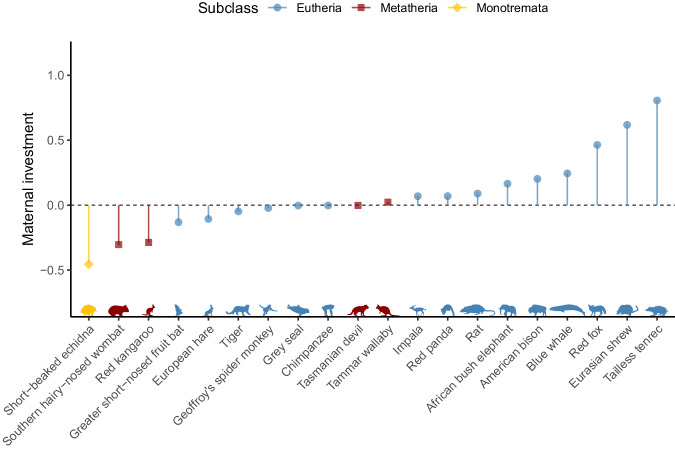
Comparison of maternal investment in twenty representative mammals. Scatter plot of the maternal investment (MI_MPLMM_) for twenty representative mammals. The horizontal dashed black line represents a maternal investment of 0, meaning that all points above this line represent a higher maternal investment than expected for an average mammal with the same default adult mass and investment duration as the species considered and all points below it represent a lower maternal investment than the same baseline. Credits for the silhouettes (from left to right): Becky Barnes, Sam Arman, T. Michael Keesey, Baheerathan Murugavel, Ferran Sayol, Gabriela Palomo-Munoz, Andy Wilson, Kai Caspar, Margot Michaud, Margot Michaud, Geoff Shaw, T. Michael Keesey, T. Michael Keesey, Ferran Sayol, T. Michael Keesey, Gabriela Palomo-Munoz, T. Michael Keesey, Rebecca Groom, Becky Barnes, Yan Wong. See legend of Table 3 for scientific names.

To illustrate the variation in maternal investment between mammals, we calculated maternal investment (*MI*_MPLMM_) for 20 indicator species belonging to three different subclasses and 13 different taxonomic orders (Table 3, Fig. 6). Since our goal here is to compare species scattered within the entire mammalian tree, we computed all *MI*_MPLMM_ values based on a single fit of the model MPLMM (for the resulting *MI* formula, see row MPLMM in Table 2). We selected these species subjectively so as to strike a good balance between retaining well-known species, including species with various characteristics, and encompassing a priori a relatively large range of maternal investment. For example, for *Eutheria* our sample includes what we thought may result in substantially different *MI* values. On the one hand, we selected the tailless tenrec (*Tenrec ecaudatus*) which has a litter size of up to 32 pups, the largest of all *Eutheria*^27^ and which also produces relatively large offspring. On the other hand, we included the greater short-nosed fruit bat, a species in which mothers give birth to a singleton twice a year^28^. For most *Eutheria*, the duration of the gestation and lactation period is roughly equal^29^_,_ whilst the lactation duration in the greater short-nosed fruit bat only takes a third of the gestation duration (42 and 120 days, respectively^30^). Our indicator species also include several *Metatheria* and *Monotremata* that we expected to fall within the lower end of the distribution of our metric.

**Table. 3:** Overview of the maternal investment (*MI*_MPLMM_) in twenty mammals: 15 Eutheria, 4 Metatheria, and 1 Monotremata

Species	Scientific name	Subclass	Order	*MI*_MPLMM_
Tailless tenrec	*Tenrec ecaudatus*	Eutheria	Afrosoricida	0.803
Eurasian shrew	*Sorex araneus*	Eutheria	Soricomorpha	0.625
Red fox	*Vulpes vulpes*	Eutheria	Carnivora	0.458
Blue whale	*Balaenoptera musculus*	Eutheria	Cetacea	0.216
American bison	*Bison bison*	Eutheria	Cetartiodactyla	0.180
African bush elephant	*Loxodonta africana*	Eutheria	Proboscidae	0.134
Rat	*Rattus rattus*	Eutheria	Rodentia	0.0915
Red panda	*Ailurus fulgens*	Eutheria	Carnivora	0.0576
Impala	*Aepyceros melampus*	Eutheria	Cetartiodactyla	0.0523
Tammar wallaby	*Macropus eugenii*	Metatheria	Diprotodontia	0.0101
Tasmanian devil	*Sarcophilus harrisii*	Metatheria	Dasyuromorphia	-0.0143
Grey seal	*Halichoerus grypus*	Eutheria	Carnivora	-0.0200
Chimpanzee	*Pan troglodytes*	Eutheria	Primates	-0.0270
Geoffroy’s spider monkey	*Ateles geoffroyi*	Eutheria	Primates	-0.0425
Tiger	*Panthera tigris*	Eutheria	Carnivora	-0.0628
European hare	*Lepus europaeus*	Eutheria	Lagomorpha	-0.110
Greater short-nosed fruit bat	*Cynopterus sphinx*	Eutheria	Chiroptera	-0.134
Red kangaroo	*Macropus rufus*	Metatheria	Diprotodontia	-0.304
Southern hairy-nosed wombat	*Lasiorhinus latifrons*	Metatheria	Diprotodontia	-0.319
Short-beaked echidna	*Tachyglossus aculeatus*	Monotremata	Monotremata	-0.464

## Discussion

How much energy, material and information organisms allocate to their offspring is extremely diverse and influenced by many factors^31,32^. Understanding variation in these investment strategies is a challenging task and many questions about the ecology and evolution of maternal investment remain unanswered (e.g. What factors influence maternal investment? How does maternal investment affect long-term fitness? How does maternal investment interact with other forms of parental care?). This lack of knowledge stems in part from the lack of a standardised approach that would allow for an effective comparison of maternal investment across species. In this study, we addressed this methodological gap by introducing a novel metric, allowing a large-scale comparison using existing data: the maternal investment metric, or *MI* for short.

To quantify maternal investment, we chose to rely on the weaning mass of offspring produced – an integrative measure of investment encompassing both gestation and lactation^33,34^. Rather than directly using the litter mass at weaning however, we rescaled this quantity so as to remove most of the influence stemming from differences in body mass and investment duration between species. A well-known methodological parallel to the *MI* metric is thus the encephalization quotient, which has also been estimated as an allometric residual in numerous studies to provide a proxy for intelligence^35^.

The measurement of *MI* implies to fit allometric relationships influencing the litter mass at weaning. Determining the best method to fit such relationships has been subject of debate^22,24,36–38^. We thus applied and compared a large set of possible methods. As we detected a strong phylogenetic signal in the data, we favoured the use of a phylogenetically-controlled model. Although investment duration scales with adult mass^5,6^ and has been shown in some taxa to be consistent with the metabolic theory of ecology^39^, the effect of investment duration appeared not to be completely absorbed by the effect of the adult mass. These results explain why we chose a phylogenetically-controlled model that accounts for both adult mass and investment duration, rather than opting for a more traditional bivariate model that would only consider the former predictor. While we favoured the most complex method (a heteroscedastic linear mixed-effects model accounting for phylogenetic inertia, which we labelled MPLMM), predictions did not differ substantially across the different methods. Therefore, simple regression methods remain acceptable to compute *MI* across mammals. In particular, the four simple methods we considered (i.e. SLR, MA, SMA and MSLR) led here to very similar results.

To our knowledge, we are the first to fit an allometric relationship using a particular heteroscedastic phylogenetic linear mixed model that includes random effects both in the modelling of the main response and of the residual variance, and therefore belongs to the class of double hierarchical generalised linear models^40^. This model (i.e. MPLMM) addresses at once multiple statistical challenges related to fitting allometric relationships. Being a multiple linear regression, the model fits the influence of quantitative predictors using straight lines, which is appropriate to estimate the scaling coefficients of the allometric relationships. Being a mixed model, MPLMM allowed us to model how much the random effects – used to describe how different species depart from a global intercept – covary as a function of the phylogenetic distance between species. Being a heteroscedastic model, the selected model did account for the fact that a standard multiple linear regression would violate the homoscedasticity assumption as the residual variance increases with the (log_10_) adult mass. Being a hierarchical model, we were able to consider as well that the residual variance also depends on the species identity through a random term. Accounting for all these specificities does exert some impact on the inferred allometric relationships and should thus lead to more reliable *MI* values.

To provide meaningful *MI* values, several specifications have to be considered beyond the choice of the statistical model used to predict the litter mass at weaning. First of all, one needs to consider which proxies of body mass to include in the data. Ideally one would consider adult mass data coming from females only since females do most of the parental investment in mammals. Unfortunately, sex-specific data are scarce for many organisms and yet ignoring the sex may sound problematic given that for 19% of mammals the sexual size dimorphism is thought to be noticeably male-biased, and for 7% of them it is female-biased^41^. Fortunately, our results show that relying on the default adult mass as a predictor for litter mass at weaning, a piece of information that is more available in the literature, does not seem to be particularly limiting in practice. This is true at least when a phylogenetic linear mixed model is used. Indeed, we found that the use of default adult mass to compute *MI* did not lead to a marked difference from the use of the females’ adult mass despite the overrepresentation of species with high sexual size dimorphism within the sample of 105 species used for the comparison (e.g. 6 species of *Pinnipedia*^42^ and 44 species of *Cercopithecidae*^43^). A possible explanation is that the ratio of the body mass of males to females remains relatively constant among closely related species within our subset. In this condition, the fitted allometric relationships should yield similar slopes irrespective of whether a default adult mass or a female adult mass is used as a predictor. This is because, in the linear model, the shift in intercept compensates for the effect of the shift in predictor values on the predicted values, resulting in predictions fairly unaffected by the choice of the adult mass proxy. Such a choice may become a more serious limitation if the metric is used for research within closely related species for which the degree of size dimorphism varies from species to species. In such a case, we strongly recommend spending effort to gather data on female adult mass and fit the allometric relationships using these data.

Irrespective of the proxy used for body mass, the quality of such data is important for trusting the metric. Indeed, the *MI* values as any allometric residuals, while valuable for understanding the relationships between biological variables^22^, demand careful consideration due to their susceptibility to reflect both biological signal and measurement errors. Hence, an unusually high or low *MI* value for a given species needs to be carefully examined, as it might be the result of such errors in the data rather than the indication of an interesting biological phenomena. Datasets on a large number of species are becoming increasingly more available but the quality of such data is not always optimal. For example, after noticing an extreme *MI* value for the walrus, we found out that the female adult mass of the walrus (*Odobenus rosmarus*), was incorrectly described to be 6.4 g, whereas the actual mass is on average 900 kg^44^. The differences in *MI* values obtained for the two alternative proxies of body mass dramatically decreased once we discarded a number of data entry inconsistencies in the Amniote database. Beyond obvious mistakes of this kind, which are relatively easy to handle automatically (see Methods), another limitation of such databases is that they tend to be based on captive populations for which data collection on life-history traits is easier. Yet, management and circumstances in captivity can affect certain parameters related to reproductive success^45^. The body mass of adult animals is often higher in captivity compared to the wild as demonstrated by the chimpanzee (*Pan troglodytes*)^46^, Iberian lynx (*Lynx pardinus*)^47^, elephant (*Elephantidae*)^48^, and vervet monkey (*Chlorocebus aethiops sabaeus*)^49^. The mass of the offspring is often higher in zoos as well^50^. As the maternal investment metric uses a ratio between body masses, the effect of higher masses in captivity is partially corrected, but data from wild-roaming populations would remain better for the study of evolutionary adaptations. Litter size might be affected by captive management as well; however, depending on the species and the institution, this influence can be both positive or negative (e.g. refs. ^45,51^). Other sampling biases may also be present in large-scale datasets. In particular, since most data is collected in captivity or for popular study species, a taxonomic bias towards larger species exists^52^.

To decrease the potential effect of taxonomic biases, one possibility is to consider a sample that is taxonomically quite diverse. For example, we relied here on a sample of 23 different mammalian orders including ≥5% of the species for 22 out of the 27 recognised mammalian orders. That being said, we recommend that the fit should ideally not include species from taxa that are too distant from the focal group of species one is interested in. This is because our results show that the parameters for the allometric relationships used to compute *MI* may vary across taxa. This variation of the allometry across taxa should not however justify fitting the underlying statistical model for the different groups separately. Indeed, *MI* values stemming from different fits of the same allometric relationship(s) are not comparable to each other. A comparison would introduce biases comparable to those introduced by performing conditional predictions (see Methods for details). Whenever sample size allows it, we therefore recommend fitting a single model using only data containing representatives of the groups that must be compared. For example, to compare the maternal investment between different orders, a single fit of the allometric relationships to the species of the different orders should be used for the scaling of the metric. Similarly, to compare maternal investment between for instance rodents, the metric should be scaled on a dataset including all the rodents to be compared. How *MI* behaves for intraspecific comparison remains to be determined, but in that case the same advice follows: a single model should be fitted on many individuals of the target species.

Even with a great statistical model and a great dataset in hand, one should not forget that *MI* suffers from the general limitation of most metrics: *MI* is only an approximation of a complex phenomenon. The proposed metric captures the direct allocation of energy to the offspring, quantified as body mass gain, but it does not include actual maternal investment that does not impact the litter mass at weaning or the investment duration. This includes several forms of parental care and protection. For example, we predicted a low *MI* value for the leopard (*Panthera pardus*), which correctly reflects that the investment duration through lactation is short in this species. However, this fails to capture that mothers do continue to invest in their offspring by hunting with and for them for many months after weaning^53^. Our metric also fails to capture the impact of pre-weaning loss. Such loss of offspring might again impact maternal investment. This latter limitation may not be severe however since the use of a correction for pre-weaning loss in previous research did not result in significant differences in the production rate of *Eutheria* and *Metatheria*^29^.

Another potential caveat of our metric is that the effect of paternal investment is not explicitly considered. Although paternal investment in mammalian species is limited, the energetic costs and opportunity costs of male care might affect reproductive strategies^54^. As long as male care corresponds to an alternative energetic contribution to the offspring, the metric we used can successfully capture variation in paternal investment when it leads to a decrease in lactation duration. Indeed, all else being equal, in species for which paternal investment is higher, the *MI* value is thus lower, reflecting energy being saved for the mother. We cannot exclude however that in some species, parental care provided by both sexes is not alternative but acts synergistically. In such a case, paternal investment would positively impact the litter mass at weaning which would lead to *MI* values that overestimate the specific contribution of the mother. Fortunately, this issue should be limited in mammals since the weaning mass, and therefore the direct nutritional investment in the offspring, has been shown to remain the same overall irrespective of the amount of paternal care^55^.

With all the aforementioned caveats in mind, the proposed metric retains its use for many possible applications. Here we employed it to compare the maternal investment of the three mammalian subclasses. Although it is well established that *Eutheria* invest more energy in their offspring during gestation compared to *Metatheria*^56^, comparisons of the total energy expenditure on reproduction are rare across the two mammalian subclasses. Our results confirmed unambiguously that many *Eutheria* do invest more energy than *Metatheria*. Importantly, our maternal investment metric remains adequate for comparing these two subclasses because it considers the litter mass at weaning and not at birth or hatching. Indeed, the importance of the two major investment periods, the gestation and lactation periods, differs substantially between the three mammalian subclasses. *Monotremata* and *Metatheria* both produce very small offspring and have a relatively short gestation and long lactation, resulting in the birth of highly altricial offspring^29,57^ relying mainly on the lactation^58,59^. *Eutheria* on the other hand have a developmental state that can vary between altricial and precocial^60^ and have an equally long gestation and lactation period.

In contrast to our results, one study has argued that the total energy expenditure was higher in *Metatheria* than in *Eutheria*^61^. We want to point out however that this previous study was only based on a comparison between one *Metatherian* and two *Eutherian* species. Given the large interspecies variation^62^ in *MI* within a subclass revealed by our study, comparisons should not be based on a small number of species if one wants to draw conclusions at such a high taxonomic level. Similarly, another study reported that no significant differences in maternal investment could be demonstrated between *Metatheria* and *Eutheria* of the same mass^63^. Given that our sample is more taxonomically diverse and around seven times larger than the one previously used, we are confident that the signal present in our data is real. Nonetheless, albeit large, our study encompasses a still small proportion of the total number of extant *Eutheria* (*N* ~ 6164) and *Metatheria* (*N* ~ 230) species^64,65^ and we encourage others to collect or assemble a larger dataset and reassess differences in *MI* metrics between taxonomic groups.

Our study also suggests that the mean maternal investment of *Monotremata* may be lower than the mean investment of *Eutheria* and *Metatheria*, although we could not test such a difference due to the lack of data on *Monotremata* (*N* = 3). This mammalian subclass consists of only two additional species of echidna (*Tachyglossidae*) which were not present in our sample. Given the similar reproductive biology between several echidna species, the chances of a similar maternal investment are however high^66^, which would confirm a lower maternal investment for *Monotremata* compared to *Eutheria* and *Metatheria*. This would be in line with the expectations for non-mammalian oviparous species investing less in their offspring compared to viviparous species^67–69^ (see also ref. ^70^), but this would not explain why we estimated an *MI* value for the platypus (*Ornithorhynchus anatinus*) much higher than the two species of echidnas, and even higher than many *Eutheria* and *Metatheria*. The argument of oviparity being associated with lower maternal investment does not however account for lactation which occurs in *Monotremata* – the only oviparous mammals. Upon an exploration of the literature to better understand our findings, we discovered that the uptake of milk by the offspring is much higher in platypuses than in echidnas. Whereas a young short-beaked echidna requires a milk intake of 10–12% of its body mass every three to five days, the platypus requires a much higher milk intake of 10–20% of its body mass every single day^71^. The total intake of solids per kilogram of body mass, and thus maternal investment in milk production, is therefore higher in the platypus^72^. This remains true even after considering that the total concentration of solids (e.g. lipids, protein, and carbohydrates) and the investment duration are somewhat lower in platypuses compared to short-beaked echidnas. This difference in milk production could therefore explain why *MI* values differ between the two families of *Monotremata* and why the *MI* value of the platypus remains high relative to many species from other mammalian subclasses.

We also compared maternal investment for twenty well known species present in the Amniote database. Although we did not have clear predictions about how high maternal investment may be for every single species, our results confirmed specific predictions. In particular, our metric shows that the tailless tenrec presents the highest maternal investment among the 738 species in agreement with being the mammal with the highest known litter size. The greater short-nosed fruit bat – a species for which the reproductive characteristics made us predict a particularly low maternal investment – also appeared to be the species with the lowest *MI* value within the 15 selected *Eutheria*. Additionally, the highest ranking *Metatherian* only reached the tenth position in decreasing order of *MI* values.

Applying the maternal investment metric methodology, it should be possible to use our *MI* metric for animal classes other than mammals. It may even be useful to study reproductive strategies in plants. Indeed, Hendriks and Mulder^73^ showed that total offspring mass per reproductive event and the adult mass scale with each other in both animals and plants. In addition, Brown et al.^74^ showed that the individual biomass production (i.e. the investment in reproduction and/or growth in ratio to the biomass of the investor per year) scales with the mass of the organism for plants, zooplankton, fish, birds, and insects. Comparing different classes of animals should be handled with care due to different reproductive strategies (such as no/limited post-partum/hatching parental care in most reptiles^75^ or extensive parental care of both males and females in most birds^76^). Additionally, large gaps in life-history data are present in non-mammalian species^77^. Some adjustment would also have to be made for computing the *MI* metric. An equivalent value for litter mass at weaning for non-mammalian species would have to be determined, such as clutch mass in birds and reptiles^78^. Additionally, an equivalent for the investment duration would have to be defined, or it would have to be dropped (i.e. the maternal investment metric would have to be based on the model PLMM rather than MPLMM).

Further research might make it possible to include more aspects of maternal investment in the metric and expand the opportunities of this approach to quantify maternal investment. Importantly, a dataset containing direct estimates of maternal investment (e.g. calorimetric maternal investment data) for multiple species would be required to further optimise and validate the *MI* metric. Despite some of the difficulties aforementioned, collecting data on body mass on a large number of species is generally easier compared to acquiring physiological data more directly reflecting maternal investment. The ease of data collection makes the established metric highly practical and applicable across a wide range of versatile scenarios.

By introducing a novel approach to quantify maternal investment, this research strives to advance our understanding of the intricate relationships associated with reproduction, offering a foundation for further exploration in the field of maternal investment and its impact on the fitness and dynamics of animal populations. It is a promising tool to further improve our understanding of maternal investment and, in a larger context, of the evolution of reproductive strategies.

## Methods

### Data collection

All life-history data were derived from the Amniote database^26^. Only data on mammalian species for which the default adult mass, weaning mass, and litter size were available (*N* = 1053) were used. Default adult masses, exceeding the highest or the lowest sex specific adult mass with more than 15% were excluded. The same was applied to weaning masses exceeding the default adult mass with more than 15%. An obvious error in walrus and sea otter data was corrected with data derived from the Encyclopedia of Marine Mammals^44^ and Walker’s Mammals of the World^79^. This resulted in a subset of 1041 species. To conduct phylogenetically-controlled analyses, we derived data from the Mammal tree^25^. A subset was generated based on species present in both the phylogenetic tree and Amniote database. Additionally, for the comparison of the six regression methods (SLR, PLMM, SMA, MA, MSLR, MPLMM), species for which the gestation duration (excluding embryonic diapause) and lactation duration were not available, were dropped, resulting in a subset of 738 species. The data from the Amniote database were mainly collected from captive animals. Unfortunately details on the source of the default adult mass were not provided in the Amniote database. A subset (*N* = 105) of species for which female adult masses were reported was created to determine the effect of sex on the predicted litter mass at weaning and consequently on the maternal investment metric.

### Statistics and reproducibility

All analyses were conducted using R version 4.3.1^80^. We provide all the R code used to produce the results and the illustrations of this paper via a repository hosted on GitHub (https://github.com/courtiol/mammalianMI). To fit the allometric relationships, we fitted six different regression models: 1) SLR, 2) PLMM, 3) SMA, 4) MA, 5) MSLR, and 6) MPLMM. We used the R packages smatr version 3.4.8^81^ to fit the models SMA and MA and we used spaMM version 4.4.16 & 4.4.23.1^82^ to fit all the other models.

The SLR, SMA and MA models all correspond to a simple bivariate linear regression of the form: ${y}_{i}=a+b\times {x}_{i}+{\epsilon }_{i}$, where in our case ${y}_{i}$ is the log_10_ of the litter mass at weaning for the species $i$, $a$ is the intercept of the linear regression, $b$ is the slope of the linear regression, ${x}_{i}$ is the log_10_ adult mass, and ${\epsilon }_{i}$ is the residual for species $i$. The exact form of ${\epsilon }_{i}$, and how $a$ and $b$ are estimated, differ between the simple linear regression (SLR), the standardised major axis regression (SMA), and the major axis regression (MA). The differences between these three methods lies in what errors the residuals are encompassing. In SLR, the residuals only capture errors in the *y*-axis, while SMA and MA are two closely related methods accounting for errors in both axes. These differences have been detailed extensively in the literature (see e.g. Warton et al.^24^ for a presentation in the context of allometry) and debates about when to use one method or the other have been going on for decades^22–24^. Irrespective of these technical differences, all three regressions yield the following allometric relationship: ${{y{{\hbox{'}}}}}_{i}={a{{\hbox{'}}}}\times {{x{{\hbox{'}}}}}_{i}^{b}$, where ${{y{{\hbox{'}}}}}_{i}$ is the litter mass at weaning for the species $i$ (without log-transformation), $a$’ equates ${10}^{a}$ (with $a,$ as defined above, the intercept of the linear regression), ${{x{{\hbox{'}}}}}_{i}$ is the adult mass (without log-transformation), and $b$ is the scaling coefficient of the allometric relationship and thus equates the slope of the underlying linear regression. The MSLR method is a simple extension of the SLR method that enabled us to consider the influence of a second predictor: the investment duration. The corresponding equations are thus ${y}_{i}=a+b\times {x}_{i}+c\times {z}_{i}+{\epsilon }_{i}$ and ${{y{{\hbox{'}}}}}_{i}={a{{\hbox{'}}}}\times {{x{{\hbox{'}}}}}_{i}^{b}\times {{z{{\hbox{'}}}}}_{i}^{c}$, where ${z}_{i}$ is the log_10_ of the investment duration, ${{z{{\hbox{'}}}}}_{i}$ the investment duration in its original scale (i.e. days) and $c$ is both the slope associated with the log_10_ investment duration in the linear regression and the (partial) scaling coefficient of the allometric relationship between investment duration and the litter mass at weaning. The meaning of the other parameters does not change from the original SLR model, except that $b$ becomes a partial scaling coefficient of the allometric relationship between the adult mass and the litter mass at weaning.

The PLMM and the MPLMM are more complex. They correspond to linear mixed-effects models, often referred as mixed-models or LMM for short. In these models, the regression equations become ${y}_{i}=a+b\times {x}_{i}+{r}_{i}+{\epsilon }_{i}$ and ${y}_{i}=a+b\times {x}_{i}+c\times {z}_{i}+{r}_{i}+{\epsilon }_{i}$ where ${r}_{i}$ is a random effect term which contributes to the departure of each species from the intercept $a$. This random term has mean 0, a variance $\lambda$ and a covariance that is a function of the phylogenetic distance between species. We used the Pagel’s correlation function^83,84^, as implemented by the function corPagel() from the R package ape version 5.7.1^85^. We initialised the correlation matrix produced by corPagel() using the R package nlme version 3.1.164^86^ and provided it to the function fitme() used to fit the model in spaMM using the argument corrMatrix.

Since we noticed heteroscedasticity in the errors of these mixed-model fits, we defined a specific residual model for the models PLMM and MPLMM. As in the models SLR and MSLR, the residuals are normally distributed and defined as ${\epsilon }_{i}=N(0,\phi )$; but while$\,\phi$ – the variance of a Gaussian distribution of the error – is a constant value in these simpler models, for the models PLMM and MPLMM,$\,\phi$ is itself a variable described by a statistical model to be fitted. Specifically, we have defined $\log (\phi )=d+e\times {\alpha }_{i}+{s}_{i}$, where $d$ is the intercept of the residual dispersion model, $e$ its slope, ${\alpha }_{i}$ the log_10_ adult body mass of the species $i$ and ${s}_{i}$ is a random gaussian term which contributes to the departure of each species from the intercept $d$. This random term has mean 0, a variance $\gamma$, but this time we considered a null covariance between the realisations of the random effects and thus did not consider the effect of the phylogenetic distance for the residual dispersion model. The models PLMM and MPLMM are thus models with two components. The first component, called mean model, models the log_10_ litter mass at weaning based on the log_10_ adult mass, the log_10_ investment duration (in the case of the MPLMM), the species identity and the phylogenetic distance of that species to the other species. The second component, called the residual dispersion model, is the model for the (log) variance of residual error of the first component, based on the log_10_ adult mass and the species identity. In spaMM, the function fitme() allows to fit jointly all the parameters indicated above (i.e.$\,a,b,c,\lambda ,d,e,\gamma$) and the models PLMM and MPLMM thus correspond to so-called double hierarchical generalised linear model^40^. There is one parameter that cannot be fitted by a single call to fitme() without further programming: the correlation parameter of the function corPagel() which is usually referred to as Pagel’s lambda (here denoted Λ). To estimate this parameter, we thus built a wrapper around the function fitme(), returning the log likelihood of the fit for fixed Λ, and maximised the likelihood, using this wrapper as objective function, using the function optimise() from R. Note however that fitme() can directly fit the model including corPagel() correlation structure with fixed Λ.

For all six regression models, we computed confidence intervals using the generic function confint() which calls a specific underlying function for each case. For the model PLMM and MPLMM, we computed the confidence intervals for all parameters but Λ by parametric bootstrap using 1000 bootstrap replicates. We computed the confidence interval for Λ using log-likelihood profiling. We compared the models SLR, PLMM and MPLMM using a likelihood ratio test for which the distribution of the test statistic under the null hypothesis was also estimated using parametric bootstrap using 1000 bootstrap replicates. For this, we used the function anova() from spaMM.

When manipulating mixed models for predicting values (in our case the predicted litter mass at weaning, which is used to obtain *MI* values), two alternative types of predictions can be computed: so-called conditional predictions which include the realisation of the random effects, and marginal predictions which consider the expected values of random effects independently of their values predicted by the fit of the model to the data. This expected value is zero in our case. The marginal predictions here reflect only the allometric relationship, while the conditional predictions include random effect values which are driven by differences in ecology and genetics that are correlated along the phylogeny. Residuals from such conditional predictions would therefore remove these ecological and genetic effects. On the contrary, residuals from the marginal predictions include them as components of the *MI* we aim to measure. These marginal species-level predictions do account for the phylogeny in the sense that the allometry relationships established for all species depend on the phylogenetic correlations (this is because fixed effects depends on random effects and vice versa), and species-level predictions remain influenced by the specificities of the species that correlate with the phylogeny.

Beyond the fit of statistical models, we also relied on a few traditional statistical parametric and non parametric tests. Specifically, we relied on Pearson correlations using the functions cor() and cor.test() to assess potential correlation between different variables. We used Quade tests to compare predicted *MI* values obtained for the different species across alternative regression methods using the function quade.test() readily available in R. We used the Wilcoxon-Pratt signed-rank test, which directly corresponds to a Quade test for comparison of two conditions only, to test whether using two alternative proxies for adult body mass had a noticeable effect in predicted *MI* values. This time, since we used a smaller dataset, we used the version of this test provided by the R package coin version 1.4.3^87^ named wilcoxsign_test(), which we set so as to provide an exact computation of the p-value. For comparisons between groups made of different species, we similarly used the Kruskal-Wallis test implemented in the function kruskal.test() available in R and the Wilcoxon-Mann-Whitney test provided by the function wilcox_test() from the R package coin.

For creating the figures, we used the R packages ggplot 2 version 3.4.4^88^, ggdist version 3.3.1^89^ which provides the geometries used to represent distributions in Figs. 4 and 5, and patchwork version 1.2.0^90^ which we used to assemble sub-figures togethers. We also used the package rphylopic version 1.3.0^91^ to handle the silhouettes of animals in Fig. 6. For reshaping datasets, we finally used the R package tidyr version 1.3.1^92^.

### Reporting summary

Further information on research design is available in the Nature Portfolio Reporting Summary linked to this article.

### Supplementary information

Reporting Summary

## Data Availability

The life history data and R code that support the findings of this study are available in an open access GitHub repository: https://github.com/courtiol/mammalianMI.
